# Contribution of Glycosaminoglycans to the Microstructural Integrity of Fibrillar and Fiber Crimps in Tendons and Ligaments

**DOI:** 10.1100/tsw.2010.192

**Published:** 2010-10-01

**Authors:** Marco Franchi, Viviana De Pasquale, Désirée Martini, Marilisa Quaranta, Maria Macciocca, Alessio Dionisi, Vittoria Ottani

**Affiliations:** Department of Anatomical Sciences, Faculty of Sport Sciences, University of Bologna, Italy

**Keywords:** tendon, ligament, fibrillar crimp, fiber crimp, glycosaminoglycans, chondroitinase ABC, electron microscopy

## Abstract

The biomechanical roles of both tendons and ligaments are fulfilled by the extracellular matrix of these tissues. In particular, tension is mainly transmitted and resisted by protein (collagen, elastin) fibers, whereas compression is opposed by water-soluble glycosaminoglycans (GAGs). GAGs spanning the interfibrillar spaces and interacting with fibrils through the interfibrillar proteoglycans also seem to play a part in transmitting and resisting tensile stresses. Both tendons and ligaments showing similar composition, but different functional roles and collagen array, exhibit periodic undulations of collagen fibers or crimps. Each crimp is composed of many knots of each single fibril or fibrillar crimps. Fibrillar and fiber crimps play a mechanical role in absorbing the initial loading during elongation of both tendons and ligaments, and in recoiling fibrils and fibers when tissues have to return to their original length. This study investigated whether GAGs covalently attached to proteoglycan core proteins directly affect the 3D microstructural integrity of fibrillar crimp regions and fiber crimps in both tendons and ligaments. Achilles tendons and medial collateral ligaments of the knee from eight female Sprague-Dawley rats (90 days old) incubated in a chondroitinase ABC solution to remove GAGs were observed under a scanning electron microscope (SEM). In addition, isolated fibrils of these tissues obtained by mechanical disruption were analyzed by a transmission electron microscope (TEM). Both Achilles tendons and medial collateral ligaments of the rats after chemical or mechanical removal of GAGs still showed crimps and fibrillar crimps comparable to tissues with a normal GAG content. All fibrils in the fibrillar crimp region always twisted leftwards, thus changing their running plane, and then sharply bent, changing their course on a new plane. These data suggest that GAGs do not affect structural integrity or fibrillar crimp functions that seem mainly related to the local fibril leftward twisting and the alternating handedness of collagen from a molecular to a supramolecular level.

## INTRODUCTION

Tendons and ligaments are connective tissues acting like passive composite dense fibrous structures that transmit loadings to bone, thus favoring joint motions and locomotion. They have a similar composition and a comparable structural array, but exhibit different functional roles: tendons efficiently transfer tensional forces generated from muscle contraction to bone and promote locomotion, while ligaments connect bone to bone guide and limit joint motions[1,2,3].

The transmission of forces within these tissues is strictly related to the composition and structural arrangement of the tendon and ligament extracellular matrix. The extracellular matrix contains fibrillar collagen ordered in a hierarchical array from a molecular to a supramolecular and organ level, and embedded in a rich amorphous matrix of glycosaminoglycans (GAGs) associated with proteoglycan proteins also containing a small quantity of other glycoproteins[4,5,6,7]. Tension is mainly transmitted and resisted by protein (collagen, elastin) fibers, whereas compression is opposed by water-soluble GAGs that also seem to play a part in transmitting and resisting tensile stresses by interacting with collagen fibrils through the interfibrillar proteoglycans[8].

The fibrillar collagen hierarchical arrangement in tendons and ligaments consists of a sort of alternating handedness, from a molecular to a macromolecular level, resembling a rope-like structure that would justify the high resistance of fibrils to tension[5,9,10,11]. Three left-handed polypeptide chains assemble into a unique triple right-handed tropocollagen helix[10,12,13,14,15]. Five tropocollagen molecules assemble, twisting in a left-handed helix, to form a single microfibril[16,17,18] and several microfibrils laterally aggregate in a right-handed helix to form a single collagen fibril[3,13,19,20,21]. When the large diameter and rigid fibrils of the tendon sharply change direction, they first twist on the left and then bend to form a flexible knot, also called *fibrillar crimp*. The left-twisted fibrils give rise to left-flattened helices observed both in isolated fibrils and sections of different tendons and ligaments. These flattened helical fibrils assemble, forming crimped fibers that align along the direction of loading and transmit tensile loads[22]. During the initial elongation of tendons, the crimped collagen fibers or crimps straighten along the direction of loading, playing a biomechanical role in absorbing shock and protecting tendon health[23,24]. However, when both tendons and ligaments have to return to their original length, crimps of collagen fibers, formed by many knots or fibrillar crimps located at the top angle of each crimp, also play a key role in recoiling both the fibrils and fibers[25].

The other components involved in fulfilling the mechanical properties of tendons and ligaments and embedded in the amorphous extracellular matrix are the proteoglycan molecules, including GAG chains[6]. Among GAGs, dermatan and chondroitin sulfate make up the majority of all sulfated GAGs in ligaments and tendons, and are the exclusive sulfated GAG chains of the interfibrillar proteoglycans decorin and biglycan. Proteoglycans contribute to the fibril and fiber structural integrity, forming interfibrillar linkages. They are highly hydrophilic in nature, thus providing for lubrication and water retention within tissues (about 70% water), and are considered the major factor controlling the viscoelastic properties of tendons and ligaments[6,7,8,26,27,28,29,30,31,32,33,34,35]. Despite these numerous studies, it was suggested more recently that decorin does not contribute to the mechanical properties of connective tissues via load transfer[36]. Moreover, Lujan et al.[37] demonstrated that the quasi-static elastic material properties of the human medial collateral ligament of the knee were unchanged after dermatan sulfate removal, challenging the theory that decorin proteoglycans may contribute to the elastic behavior of the ligament. Ligament viscoelasticity did not change after the complete removal of all GAGs in the medial collateral ligament[38].

These inconsistent data prompted us to investigate whether GAGs, mainly GAGs covalently attached to interfibrillar proteoglycan core proteins, directly affect the 3D microstructural integrity of fibrillar crimp regions and fiber crimps in both tendons and ligaments.

## MATERIALS AND METHODS

Eight female Sprague-Dawley rats (90 days old) were anesthetized with an intraperitoneal injection of 87 mg/kg ketamine (Ketavet; Farmaceutici Gellini S.p.A., Italy) and 13 mg/kg intramuscular xylazine (Rompun; Bayer Italia S.p.A., Italy). After skin incision, the Achilles tendons and medial collateral ligaments of both posterior legs were carefully exposed by dissecting away the overlying fascia and then were excised at the ends. The rats were then killed via an intracardiac injection of Tanax (Hoechst, Frankfurt am Main, Germany).

Six Achilles tendons and six medial collateral ligaments were immediately incubated in a cacodylate buffer solution + chondroitinase ABC from *Proteus vulgaris* (ChABC) (Sigma-Aldrich, Milano, Italy) for 6 h. After this period, the specimens were fixed in a Karnovsky's solution (4% paraformaldehyde + 2.5% glutaraldehyde in cacodylate buffer 0.1 M, pH 7.4) for 24 h, postfixed in 1% osmium tetroxide, dehydrated in a graded series of alcohol, sectioned following a sagittal plane, and finally dehydrated in hexamethyldisilazane. The sectioned specimens were then mounted on metal stubs and coated with 5 nm gold in an Emitech K550 sputter-coater to be observed under SEM (Philips 515, Eindhoven, The Netherlands) operating in secondary-electron mode.

Two Achilles tendons and two medial collateral ligaments were finely chopped with razor blades immediately after dissection and then subjected to a gentle mechanical disruption with a Dounce homogenizer. Drops of the suspension containing isolated collagen fibrils were put on Formvar-coated, 300-mesh copper grids. Negative staining was carried out by dripping 1% phosphotungstic acid dissolved in 0.1 M phosphate buffer, pH 7.4. Double positive staining was performed by putting grids on drops of 1% phosphotungstic acid aqueous solution, pH 3.2, for 10 min and, after brief washing in distilled water, on drops of 1% uranyl acetate aqueous solutions, pH 4.2, for 20 min. The grids were subsequently washed in distilled water and dried with filter paper. Specimens were examined under TEM (Philips CM-10, Eindhoven, The Netherlands).

All the procedures were performed strictly following Italian and European Law on animal experimentation.

## RESULTS

After removal of GAGs with ChABC, the sectioned Achilles tendons and medial collateral ligaments of the rats showed, in general, regularly packed collagen fibers mainly running almost straight and parallel, but interrupted by occasional undulations or crimps of different sizes. However, medial collateral ligaments showed regions with collagen fibrils and fibers more cross-connecting to each other and more intertwined than in the tendons (Figs. 1–3).

All collagen fibers appeared composed of densely packed fibrils showing a diameter of about 30–170 nm. At the apex of each fiber crimp, each single fibril sharply bent, showing a different size of diameter and a particular local structural morphology previously described as a fibrillar knot or fibrillar crimp. In particular, each fibril in the fibrillar crimp region first twisted to the left (about 180°), changing the plane of direction, and then sharply bent, changing the direction of its course (Figs. 3, 4). Some fibrils also showed two or more very close knots within a single fiber crimp.

The isolated collagen fibrils from Achilles tendons and medial collateral ligaments of the rats observed at TEM generally showed a straight course and a regular D-banding of about 67 nm. Some of them that suddenly changed direction at the top of each fiber crimp appeared squeezed and exhibited the typical knots or fibrillar crimps. At in-depth observation in the fibrillar crimp region, the collagen fibrils always twisted to the left, changing their running plane, and then bent, changing their direction. In some fibrillar crimp regions, the fibrils showed an irregular D-banding, but sometimes they seemed to lose this pattern, also showing an apparent local microfibrillar swelling (Fig. 5).

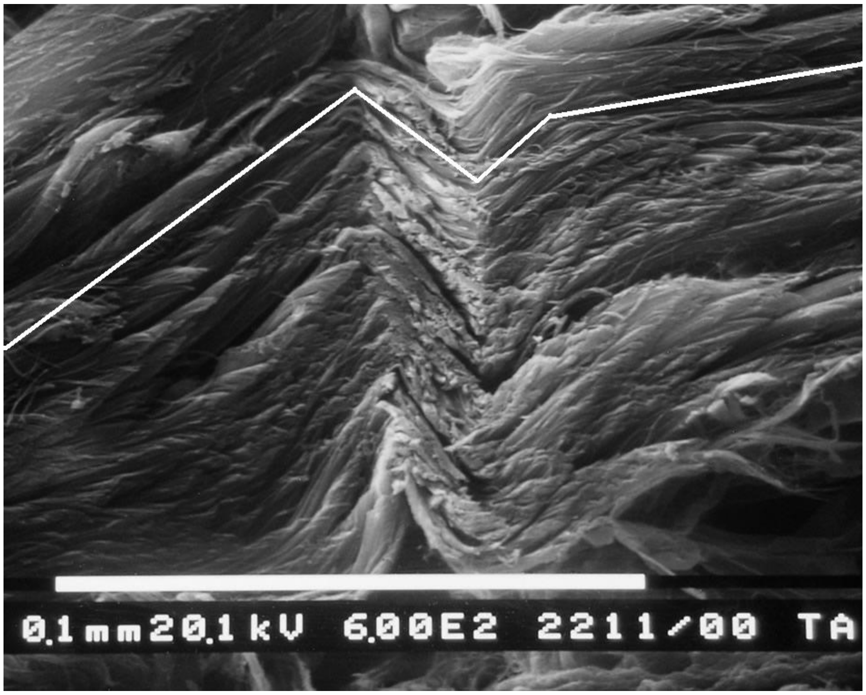

**FIGURE 1.** Rat Achilles tendon incubated in ChABC observed at SEM. Densely packed collagen fibers running almost straight and parallel appear interrupted by zig-zag undulations or crimps (indicated by the white line). Bar = 100 µm.

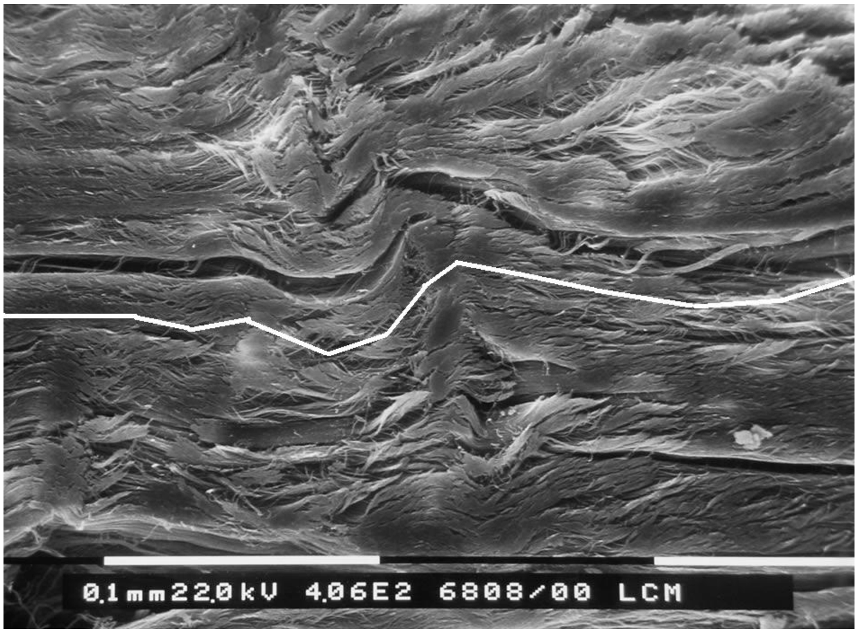

**FIGURE 2.** Rat medial collateral ligament incubated in ChABC observed at SEM. Densely packed collagen fibers mainly running almost straight and parallel show occasional undulations or crimps of different sizes. A cross-connection of collagen fibers can also be seen (indicated by the white line). Bar = 100 µm.

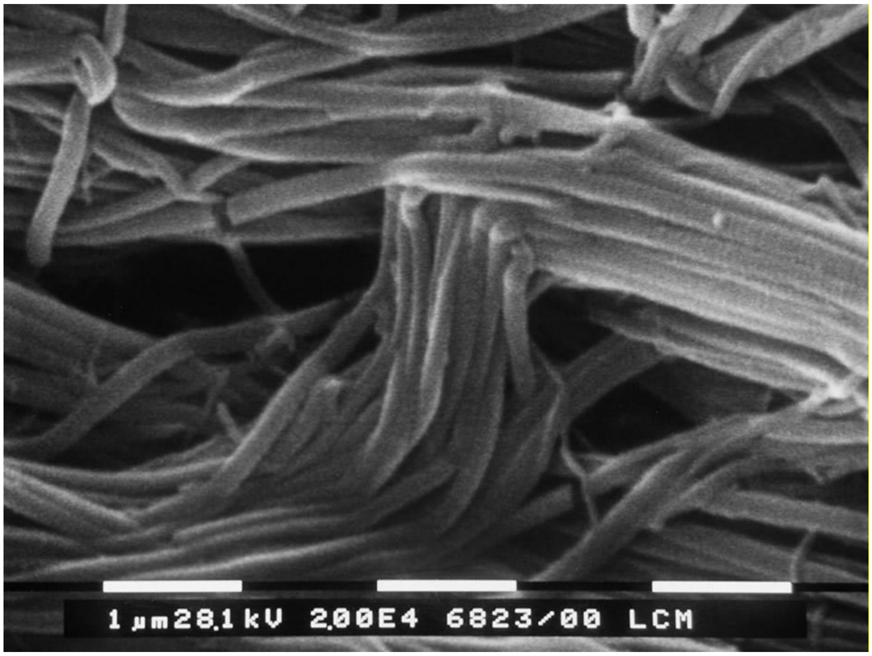

**FIGURE 3.** Rat medial collateral ligament incubated in ChAB observed at SEM. At higher magnification in the fibrillar crimp region, fibrils show a cross-connection to each other. Each fibril forming a knot or fibrillar crimp clearly first twists to the left (about 180°), thus changing the plane of direction, and then sharply bends, modifying the direction of its course. Bar = 1 µm.

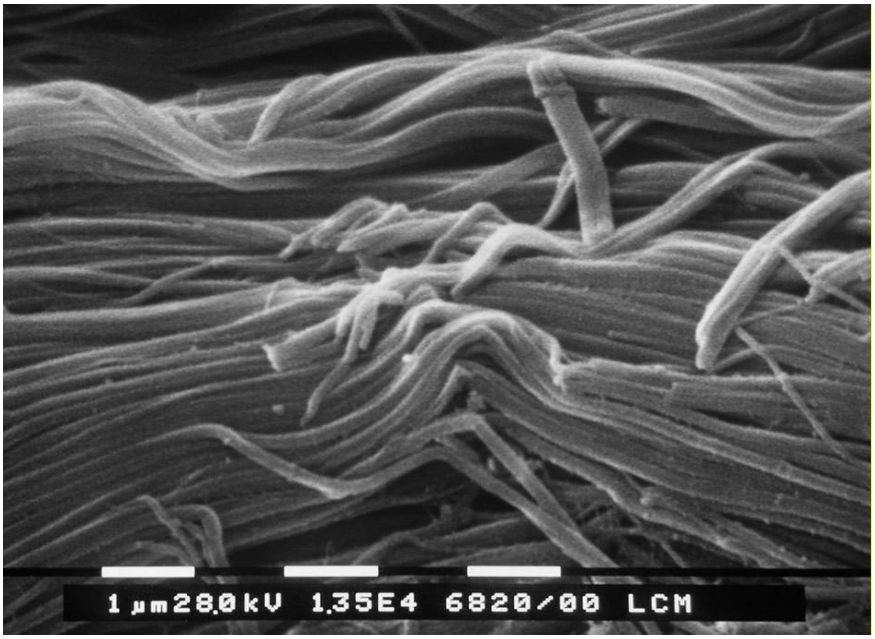

**FIGURE 4.** Rat medial collateral ligament incubated in ChABC observed at SEM. In the fibrillar crimp region, each fibril first twists on the left, changing the plane of direction, and then sharply bends, changing the direction of its course. Bar = 1 µm.

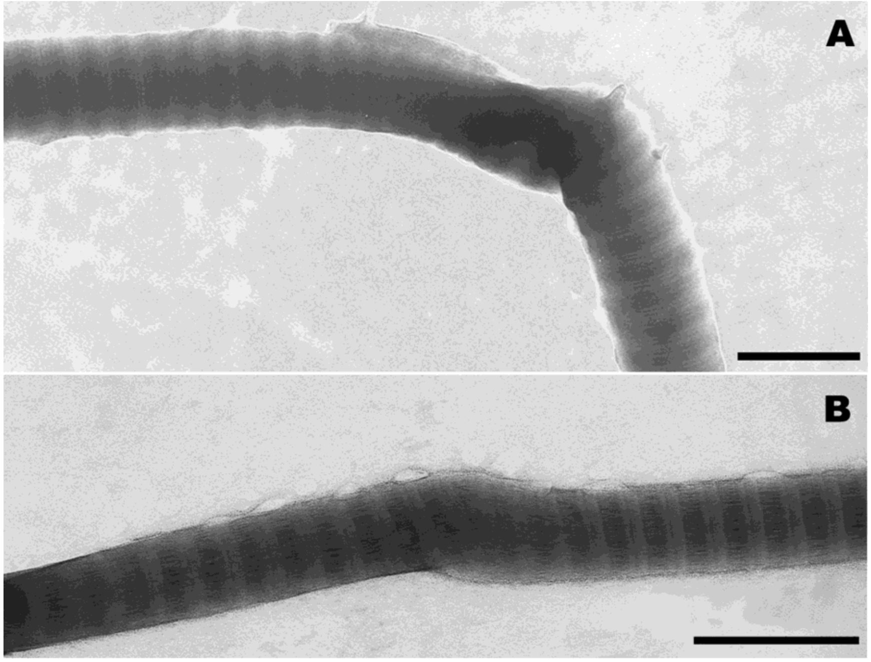

**FIGURE 5**. (A) An isolated fibril from rat Achilles tendon observed at TEM. The fibril shows a knot comparable to a fibrillar crimp. In the fibrillar crimp region, the fibril twists to the left and then sharply bends. The periodic D-banding of about 67 nm appears less recognizable in the fibrillar crimp region. (B) An isolated fibril from rat medial collateral ligament observed at TEM shows a straightened fibrillar crimp. In the fibrillar crimp region, the periodic D-banding appears less regular and a microfibrillar swelling is partially evident. Bar = 200 nm.

## DISCUSSION

Tendons and ligaments are dense connective tissues with a similar composition, but play very different mechanical roles and consequently show a different collagen array. An Achilles tendon transmitting mainly tensional forces along the course of its collagen fibers during muscle contraction shows straight and parallel densely packed fibers, whereas the medial collateral ligament submitted both to tensional and rotational forces shows both straight and parallel, and also very intertwined, fibers[39]. Despite their structural and functional differences, tendons and ligaments both show a crimping pattern or periodic crimps along their collagen fibers. Tendon and ligament crimps act as a protection system or mechanical buffer, absorbing the initial tensional load and recoiling the collagen fibers and fibrils after loading[22,25,40,41]. In the stress-strain curve of the tendon, the initial toe region corresponds to the straightening of crimps. Then the tendon exhibits a linear region of elongation that involves either extension of the individual collagen components or relative sliding between them[35,42]. In particular, sincrotone X-ray diffraction studies of the tendon matrix demonstrated that only 40% of tendon elongation occurs at the molecular level, whereas about 60% of tendon extension is due to sliding between one or more of the structural levels of collagen[43,44,45]. Interfibrillar sliding is related to the interfibrillar proteoglycans. Dermatan sulfate is the most abundant component of GAGs in the extracellular matrix of the tendon and is covalently attached to the core protein of decorin and biglycan proteoglycans; dermatan sulfated GAGs span the space between neighboring collagen fibrils, thus creating interfibrillar functional “cross-links” and are preferentially distributed orthogonally to the fibril direction[32,46,47]. It has been suggested that dermatan sulfated GAGs could transfer forces between discontinuous fibrils, thereby playing a functional role during tendon elongation[6,7,26,27,28, 29,30,31,32,33,34]. Decorin is a regulator of collagen fibril structure because it limits collagen fibril formation and contributes to the structural array of fibrils in tendon and ligaments[48].

We investigated whether GAGs affect the 3D structural array of fiber and fibrillar crimps, and could play a role in tension absorbing/fiber recoiling in both tendons and ligaments. After enzymatic degradation of GAGs by ChABC incubation, and mechanical disruption of collagen fibers and fibrils in the Achilles tendon and medial collateral ligament, crimps composed by fibrillar knots or fibrillar crimps were still observed. They showed the original morphological structure previously seen in tissues with a normal GAG content. ChABC removes the interfibrillar GAG chondroitin and dermatan sulfate, but also degrades hyaluronic acid, a large unsulfated GAG noncovalently associated with aggrecan and chemically similar to chondroitin sulfate[38].

This study demonstrated that GAGs and proteoglycans do not affect the structure of fibers and fibrillar crimps, and probably do not play a direct role in fibrillar crimp function, such as shock absorption and collagen fiber and fibril recoiling. These results are in agreement with data reporting that GAGs do not contribute to either the quasi-static tensile-shear material properties or viscoelastic tensile behavior of the human medial collateral ligament[37,38]. Lewis et al.[36] also refuted the notion that decorin contributes to the mechanical properties via load transfer in tendons. Moreover Screen et al.[35] reported that different environments can influence the swelling of collagen fibers and fibrils, and change the results of mechanical testing on tendons, but the strain at which crimps are fully straightened is always the same. Our observations and the data reported by these authors suggest that the fibrillar crimp structural array (fibrillar knot) seems to be related to the particular local left twisting of the single fibril and to the hierarchical alternated handedness of collagen from a molecular to a supramolecular level. The hierarchical arrangement of collagen molecules makes the fibril resemble a rope-like structure[9,10]. In the fibrillar crimp region, both fibrils of tissues from where GAGs were chemically removed and isolated fibrils obtained by mechanical disruption always twist leftwards, changing their running plane, and then sharply bend, changing their course on a new plane. A repetitive left-handed twisting of each fibril gives rise to left-handed fibril helices acting as biological hinges, both absorbing tension during fibril strengthening and guiding the recoil of collagen fibers when tension is removed[22].

## ACKNOWLEDGMENTS

The authors thank Gianfranco Filippini, Dipartimento di Scienze e Tecnologie Agroambientali, University of Bologna, for his technical assistance with SEM. This work was supported by MIUR grant (Ricerca Fondamentale Orientata 2007).
